# Patent Foramen Ovale (PFO) Closure in Thrombophilia as Primary Prevention in Patients Without Prior PFO-Associated Thromboembolic Events

**DOI:** 10.7759/cureus.88492

**Published:** 2025-07-22

**Authors:** Anna Maina, Nikolaos Ziogas, Andreas Sarantopoulos, Ioannis Karamatzanis, Panagiota Kosmidou, Konstantinos Mainas, Aphrodite Tzifa

**Affiliations:** 1 Medicine, European University Cyprus, Nicosia, CYP; 2 Internal Medicine, Ipswich Hospital, Ipswich, GBR; 3 Otolaryngology - Head and Neck Surgery, Mediterranean Hospital of Cyprus, Limassol, CYP; 4 Otolaryngology - Head and Neck Surgery, University of Patras, Medical School, Patras, GRC; 5 Cardiology, Private Practice, Komotini, GRC; 6 Pediatric Cardiology, Mitera Children’s Hospital, Athens, GRC

**Keywords:** patent foramen ovale, percutaneous interventions, pfo, pfo closure, thrombophilia

## Abstract

Patent foramen ovale (PFO) is a common anatomical variant with potential clinical implications in embolic phenomena. In some situations, transcatheter closure is considered a treatment option. For patients with thrombophilia who have not had embolic events, it is not always clear whether this approach is appropriate. Individuals with clotting disorders may face different considerations, and approaches can vary between cases. This study was conducted based on the PICO-formatted research question: Population - thrombophilic patients with no history of embolic events; Intervention - PFO closure; Comparison - medical therapy; Outcome - stroke prevention. We focused on asymptomatic individuals due to their theoretical but unproven elevated risk of stroke and the current absence of specific guidelines for primary prevention.

Studies addressing PFO screening and management in thrombophilic patients without a prior history of stroke or embolic events were included. English-language articles published up to December 2024 were reviewed using data extracted from MEDLINE, Cochrane Library, and Scopus. The included studies primarily consisted of observational designs, such as prospective and retrospective cohort studies, case-control studies, and case series, which collectively provided the evidence base for evaluating PFO management strategies in patients with thrombophilia.

After full-text screening, only one retrospective cohort study met our inclusion criteria. This study of 136 thrombophilic patients with PFO, all maintained on antithrombotic therapy, found that primary percutaneous PFO closure was independently associated with a nearly five-fold reduction in the risk of cerebrovascular accident or transient ischemic attack (adjusted hazard ratio 0.18; 95% CI: 0.03-0.3; p = 0.001), demonstrating a statistically significant benefit of closure in this high-risk population. Although this result suggests a potential benefit of early intervention, the evidence remains limited and non-randomized. These findings should be viewed as hypothesis-generating, highlighting the need for prospective studies to better define the role of PFO closure in this specific population and to inform future guideline development.

## Introduction and background

Patent foramen ovale (PFO) is a common anatomical variant, with a prevalence estimated between 20% and 34% [1]. After birth, the incomplete fusion of the septum primum and septum secundum leads to the persistence of PFO, which serves as a residual atrial communication and may act as a conduit for paradoxical embolization [1,2]. 

PFO is detected in approximately 40-45% of patients with cryptogenic stroke [3]. To determine whether a cryptogenic stroke is likely attributable to an associated PFO, rather than an incidental finding, the Risk of Paradoxical Embolism (RoPE) score was developed. This 10-point scoring system offers a predictive framework to guide decisions regarding PFO closure without assessing the factor of thrombophilia as a potential risk of stroke recurrence [3].

Thrombophilia, or any inherited or acquired hypercoagulable state, is linked to arterial or venous thromboembolic events [4]. Inherited causes include prothrombin gene mutation, factor V Leiden mutation, deficiencies in antithrombin, protein S and C and acquired causes, such as the antiphospholipid syndrome. Patients with venous thromboembolism and PFO are at increased risk for systemic embolism and subsequent PFO-associated stroke [5-7]. The underlying pathophysiology includes paradoxical embolism or in situ thrombus formation within the PFO [8]. Coexistence of PFO and thrombophilia has been reported in 5% to 31% of patients, and one case-control study by Pezzini et al. found a significant association between the prothrombin G20210A mutation and PFO-associated stroke, compared to both non-PFO stroke patients and healthy controls [4,5,7].

The therapeutic approach to PFO was debated for a long time, particularly concerning the effectiveness of percutaneous PFO closure compared to medical therapy with antiplatelets and anticoagulants used for the prevention of recurrence. The CLOSE, RESPECT, REDUCE, PC, and CLOSURE I trials (n = 3,440) demonstrated that percutaneous PFO closure significantly reduced the risk of recurrent ischemic stroke compared to medical therapy alone [9]. The above findings were further supported [2], concluding that percutaneous PFO closure is more effective than medical therapy in reducing recurrent event rates. However, percutaneous PFO closure was associated with a significantly higher incidence of atrial fibrillation compared to medical treatment [10]. This event has a peak incidence on the 14th day after PFO closure. Mostly, it is considered benign, being spontaneously resolved after the 45th post-closure day. For this reason, a multi-disciplinary team approach (hematologist/cardiologist) evaluating the bleeding versus the stroke risk should be recommended [11].

Eventually, the American Heart Association/American Stroke Association (AHA/ASA) produced guidelines recommending PFO closure in cases of patients aged 18-60 years with a non-lacunar ischemic stroke of undetermined cause and a PFO with high-risk anatomical features such as a large shunt, atrial septal aneurysm, or hypermobility of the septum (Class 2a) [12]. In contrast, they stated that for patients in the same age group and clinical scenario but without high-risk anatomical features, the benefit of PFO closure is not well established (Class 2b), and its efficacy compared to warfarin therapy remains unknown [12].

Currently, there are no clear guidelines [5,8] for managing the subpopulation exhibiting thrombophilia, partly due to the exclusion of such patients from most randomised clinical trials [5]. The European Society of Cardiology (ESC) guidelines [8] recommend PFO closure in the presence of deep vein thrombosis and/or pulmonary embolism, but only when temporary oral anticoagulation (OAC) therapy is required or when there is a high risk of recurrence despite long-term OAC therapy. The American Academy of Neurology (AAN) [13] suggests that clinicians should neither definitively endorse nor reject PFO closure in these patients. Furthermore, thrombophilia screening is not universally recommended for all PFO patients but is limited to those under 55 years old with cryptogenic stroke [5,8]. Despite the limited data for this subpopulation, a meta-analysis by Hviid et al. [6] indicated a higher risk of stroke recurrence after an initial PFO-associated stroke. A cohort study by Liu et al. [14] showed that patients with cryptogenic embolism and thrombophilia have a higher risk of TIA/stroke recurrence and respond well to PFO closure. 

Importantly, patients with PFO and thrombophilia who have not yet experienced a cerebrovascular event represent a clinically relevant but under-investigated group. While early intervention in this population may have meaningful implications for stroke prevention, there is ongoing uncertainty and controversy regarding the role of primary prevention in the absence of prior thromboembolic events. This lack of consensus is further reflected in the absence of specific guideline recommendations, highlighting the need for individualized decision-making. Nevertheless, this population remains clinically important due to their elevated theoretical risk, and understanding the potential impact of intervention is essential.

Given the paucity of randomized data on this subgroup, this commentary review addresses a clinically important gap in stroke prevention.

## Review

Methods 

Search Strategy and Selection Criteria 

All eligible English-language articles published up to December 2024 were identified, collected, and examined in full text by two independent researchers. The initial search was conducted in three major electronic databases (MEDLINE/PubMed, Cochrane Library, and Scopus), using combinations of keywords and Boolean operators, including “Patent Foramen Ovale” OR “PFO” AND “Thrombophilia” AND “Closure” OR “PFO closure.” To enhance the detection of relevant articles, multiple advanced searches were performed in each database using combinations of the aforementioned keywords. Additionally, the references of the eligible full-text articles identified during the initial database search were manually checked for potential additional undetected reports. This study was conducted based on the PICO-formatted research question: Population - thrombophilic patients with no history of embolic events; Intervention - PFO closure; Comparison - medical therapy; Outcome - stroke prevention. Inclusion criteria comprised original research articles involving human subjects that evaluated patients with both PFO and thrombophilia, focusing on clinical outcomes related to stroke or thromboembolism, and reporting patient demographics and outcomes. The exclusion criteria encompassed studies unrelated to the topic, manuscripts published in languages other than English, and case reports, as well as systematic reviews and meta-analyses. 

Data Extraction and Risk of Bias Assessment

A standardized protocol was followed for study selection and data extraction. Discrepancies between reviewers were resolved through discussion and, when necessary, adjudicated by a third researcher. The process ensured consistent application of the eligibility criteria and minimized selection bias.

Results 

Study Identification and Screening Process

**Figure 1:**
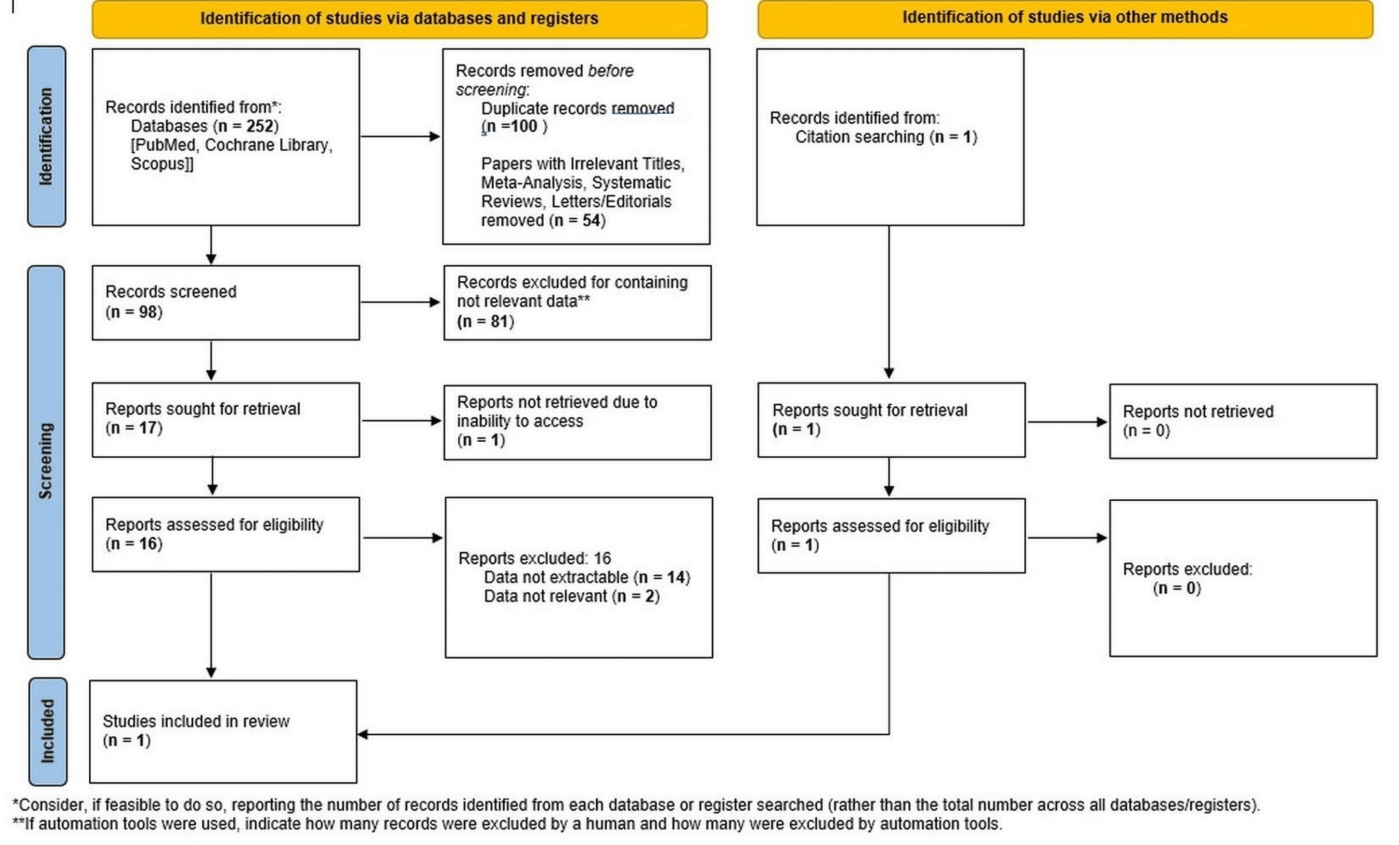
Flow diagram depicting the inclusion process of the studies identified in the searched databases according to the Preferred Reporting Items for Systematic Reviews and Meta-Analyses-Protocol (PRISMA-P) guideline

Data extraction revealed a gap in knowledge and controversial management for patients with thrombophilia. The literature selection process is depicted in detail in Figure 1, according to the Preferred Reporting Items for Systematic Reviews and Meta-Analyses-Protocol (PRISMA-P) guideline [15]. Although a large number of studies that mentioned the detection of a PFO in thrombophilia patients after a cryptogenic stroke or other embolic events were identified, there is a significant absence of literature focusing on PFO screening and prophylactic treatment in thrombophilia patients without any prior associated event. As stated in Figure 1, a study by Buber et al. [16] solely addressed this question, along with two case reports. The latter were not included in the predefined eligibility criteria for evidence synthesis but were selectively referenced in the discussion to aid in the interpretation of findings and to provide additional clinical context.

Characteristics and Main Findings of the Included Study

The sole included study by Buber et al. [16] was a retrospective cohort study that evaluated the effect of PFO closure in 136 patients with clinically significant hypercoagulable states (HCSs) who were maintained on long-term anticoagulant or antiplatelet therapy. The study spanned a mean follow-up of 46 ± 8 months. Patients were divided into two groups based on whether they underwent transcatheter PFO closure (n = 85) or not (n = 51). The primary outcome of the included study indicated that PFO closure was independently associated with a substantial reduction in the risk of cerebrovascular accidents (CVAs) or transient ischemic attack (TIA), corresponding to an approximate five-fold risk reduction (adjusted hazard ratio: 0.18; 95% CI: 0.03-0.3; p = 0.001), demonstrating strong statistical evidence. Additionally, anticoagulation therapy was associated with a statistically significant two-fold reduction in risk compared to antiplatelet therapy (adjusted hazard ratio: 0.47; 95% CI: 0.06-0.9; p = 0.05).

There is a gap in knowledge and agreed management for patients with thrombophilia and PFO. As stated in Figure 1, one study has only addressed this question. 

Discussion****

Overview of Current Evidence in Asymptomatic PFO and Thrombophilia

The management of patients with PFO and thrombophilia remains challenging due to a lack of robust data. This subpopulation includes patients who have not yet experienced a cerebrovascular event, making decisions about PFO closure particularly complex. A cohort study by Buber et al. [16] aimed to assess the impact of primary percutaneous PFO closure in patients with a clinically significant HCS who are at high risk for CVAs or TIA. The study evaluated 136 patients with HCS who were maintained on antithrombotic therapy, with 85 of them undergoing PFO closure. Notably, the study excluded patients with a prior stroke history, setting it apart from other analyses. Over a mean follow-up of 46 ± 8 months, 23 patients (17%) experienced a CVA/TIA, a rate higher than in other large PFO trials. Among these 23 patients, 16 had not undergone PFO closure, highlighting the protective effect of the procedure. Additionally, the majority of the 23 patients with outcome events had protein C deficiency. It was particularly important to note that over half of the stroke events occurred within one month of interrupting anticoagulation for medical procedures, and 85% of these events occurred in patients who had not undergone PFO closure. This finding further supports the advantage of PFO closure over antithrombotic therapy alone. A multivariate Cox proportional hazards analysis revealed that PFO closure was associated with a five-fold reduction in the risk of CVA or TIA (adjusted hazard ratio 0.18; 95% CI: 0.03-0.3; p = 0.001), independent of the type of HCS or antithrombotic therapy. Anticoagulation therapy was also protective compared to antiplatelet therapy (adjusted HR: 0.47; 95% CI: 0.06-0.9; p = 0.05), though to a lesser extent. These findings suggested that in patients with hypercoagulable states, PFO closure may offer stronger independent protection than pharmacological therapy alone. However, the study had a limited number of outcome events and potential selection and referral biases due to the lack of patient randomisation into PFO closure and antithrombotic treatment groups. In conclusion, Buber et al. [16] provided evidence supporting PFO closure as a primary prevention strategy for reducing the risk of thromboembolic events. 

Rare Clinical Cases: Insights and Hypotheses

In a separate case, an eight-year-old girl who suffered an acute myocardial infarction was found to be homozygous for the prothrombin G20210A mutation, which is associated with an increased risk of venous thromboembolism [17]. After ruling out other causes of myocardial infarction in children, such as coronary artery disease, echocardiography revealed a PFO. Carano et al. [17] suggested that the myocardial infarction was likely caused by paradoxical embolism through the PFO into the coronary arteries, a mechanism that, although rare, has been extensively described in the literature for cryptogenic stroke in young adults with PFO. The patient underwent PFO closure with a 25mm Amplatzer PFO device, followed by six months of warfarin anticoagulation therapy. This case illustrates the broader relevance of paradoxical embolism beyond cerebral circulation and into coronary vasculature, especially in thrombophilic children. Despite instances of myocardial infarction caused by paradoxical embolism through a PFO, current guidelines do not address the management of these patients, as they do for those with cryptogenic stroke and PFO. A similar case report by Croft [18] detailed a 44-year-old non-pregnant woman who experienced an acute myocardial infarction secondary to paradoxical embolism with heterozygous factor V Leiden deficiency. A PFO shunt, coexisting with an atrial septal aneurysm (ASA), was detected; hence, closure was performed. To this end, Croft [18] suggested surgical management of PFO/ASA in cases of paradoxical embolization causing acute myocardial infarction to reduce the risk of future embolic events. Although these cases illustrate the potential pathophysiological mechanism of paradoxical embolism beyond stroke, their findings are hypothesis-generating and should be interpreted carefully.

Inherited Thrombophilias and PFO: Pathophysiological Links

Consistent with these findings, genetic predispositions such as the Factor V Leiden and prothrombin G20210A mutations have been implicated in the pathophysiology of paradoxical embolism among individuals with PFO. A meta-analysis conducted by Pezzini et al. identified a significant association between the prothrombin G20210A mutation and PFO-related cerebral ischemia, with an odds ratio of 3.85 compared to healthy controls and 2.31 relative to stroke patients without a PFO. In contrast, the Factor V Leiden mutation did not demonstrate a statistically significant association, indicating a potentially less prominent role in this clinical context [7]. Complementary evidence from Karttunen et al. demonstrated that either the Factor V Leiden or the prothrombin G20210A mutation was independently associated with cryptogenic stroke in patients with PFO, yielding an odds ratio of 2.8, whereas other thrombophilic abnormalities did not show similar correlations [19]. These cumulative findings point toward a gene-specific influence on paradoxical embolic risk, which could inform more individualized decision-making regarding thrombophilia testing and prophylactic interventions.

In addition to the primary focus of this study, thrombophilia screening recommendations were also evaluated. Neither the ESC [8] and AHA/ASA [12] surveys nor guidelines do not support routine thrombophilia screening in stroke patients with PFO. An older EAPCI position paper of 2019 considers that thrombophilia screening is not warranted to indicate permanent OAC treatment (level of evidence C) [20]. However, a recent meta-analysis by Lim et al. [21] underlined the screening for thrombophilia in stroke PFO patients to be beneficial. The study found an increased risk of stroke recurrence in patients with thrombophilia, compared to those without, after PFO closure. These findings further enhanced the recommendation for thrombophilia screening in this high-risk population. 

Guideline Discordance and Evidence Limitations

Considering the findings of Buber et al. [16] and the two similar case reports [17,18], the issue of under-reporting becomes evident. The only available data are based on retrospective studies that do not account for any confounding factors for the association between inherited thrombophilia and patients with cryptogenic stroke and PFO [20]. Of interest, the 2022 Society for Cardiovascular Angiography and Interventions (SCAI) guidelines recommended against PFO closure in patients with thrombophilia without prior PFO-associated stroke [22]. This recommendation was based on the findings of the cohort study by Buber et al. [16], despite the conclusion of the study suggesting PFO closure to be beneficial in these particular patients. Of note, the SCAI guidelines committee based its recommendation on a critical appraisal of the available evidence, which was deemed to be of very low certainty due to methodological limitations, including the observational nature of the data, potential confounding, and the fragility of effect estimates. This apparent discrepancy between data interpretation and guideline formulation reflects the urgent need for high-quality, prospective studies in this subgroup.

Additionally, a cohort study by Badea et al. [23] compared patients with PFO and thrombophilia who had experienced a cerebrovascular event to those who had not. Their findings indicated that thrombophilic abnormalities such as Factor V Leiden, MTHFR mutations, or Protein C/S deficiency did not significantly increase the risk of a first stroke in PFO carriers. This suggested that thrombophilia alone may not be a sufficient criterion for guiding primary prevention strategies in asymptomatic individuals. These conclusions stand in contrast to the findings of Buber et al. [16], maybe due to differences in study populations, definitions of thrombophilia, or outcome ascertainment. For instance, Badea et al. [23] included only patients who had already experienced cerebrovascular events, whereas Buber et al. focused exclusively on primary prevention [16]. Such methodological differences may explain the divergent conclusions and emphasize the need for better-standardized research designs.

Economic Considerations: Cost Versus Evidence

Stroke currently ranks as the fourth leading cause of disability-adjusted life years (DALYs), as reported by the Global Burden of Disease Study 2021 [24]. Moreover, collected data from a national stroke registry covering England, Wales, and Northern Ireland highlight a five-fold variation in patient-related healthcare costs, ranging from £19,101 to £107,336 [25]. These financial disparities are also echoed in a population-based economic analysis across Europe, revealing that stroke survivors required approximately €1.3 billion hours of informal care, corresponding to a societal cost of nearly €16 billion [26]. Considering these findings, implementing primary prevention strategies in patients with thrombophilia could play a pivotal role in mitigating the economic burden imposed on healthcare systems. However, even though economic data underscore the substantial healthcare burden of stroke, extrapolating these findings to support primary PFO closure in asymptomatic thrombophilic patients remains premature. The cost-effectiveness of such an approach is unclear in the absence of randomized evidence demonstrating benefit. In this context, anticoagulation remains a less invasive, guideline-supported option for secondary prevention and potentially for select high-risk individuals in the primary prevention setting. Comparative studies are needed to determine whether closure offers additional value beyond medical therapy in this subgroup.

Recommendations and Future Directions

The study has significant limitations because of the lack of studies found in the literature. Further studies with larger sample sizes and uniform reporting are required. Based on the above lack of data, the present commentary review recommends adopting a more systematic and consistent approach to define the criteria that will identify patients with prothrombotic or hypercoagulable states and coexisting PFO, before they even develop cerebrovascular events. Moreover, future research should focus on establishing systematic criteria to identify patients at the highest risk who may derive the greatest benefit from early intervention. Potential candidate variables include specific genetic markers (e.g., homozygous prothrombin G20210A), PFO morphology (e.g., large shunt or atrial septal aneurysm), coagulation profiles, and clinical indicators like a history of venous thromboembolism or elevated RoPE scores. Clearly defining these parameters could facilitate the development of risk stratification models to inform clinical decision-making and ensure more efficient use of healthcare resources.

## Conclusions

The management of PFO in thrombophilia patients remains difficult and understudied, especially for those who have not yet experienced a cerebrovascular event. Despite some indications that percutaneous PFO closure may lower the risk of stroke in high-risk thrombophilic individuals, the present data are limited by underreporting and methodological limitations. Notably, this clinical ambiguity places both physicians and patients in a position of uncertainty when weighing the benefits and risks of closure versus conservative management. To overcome these problems, more focused research and clinical trials should be designed to evaluate the appropriate PFO management in this high-risk population, leading to more precise data and optimization of patient care. To address these gaps, well-designed randomized controlled trials and prospective cohort studies are urgently needed to evaluate the comparative effectiveness of PFO closure versus antithrombotic therapy in high-risk thrombophilic patients before any cerebrovascular event. Key outcomes should include the prevention of first-time stroke or transient ischemic attack, bleeding risks, and quality of life, with subgroup analyses for asymptomatic individuals. However, trials in asymptomatic populations face significant ethical and logistical challenges that have limited their conduct to date. Until high-quality prospective trials are conducted, individualized decision-making through multidisciplinary evaluation remains essential.
